# The complete chloroplast genome of *Ulmus parvifolia*, an important landscaping tree

**DOI:** 10.1080/23802359.2020.1797586

**Published:** 2020-07-30

**Authors:** Yunzhou Lyu, Min Zhai, Zeping Jiang, Qingsheng Chen

**Affiliations:** a Jiangsu Academy of Forestry, Nanjing, China; b Institute of Botany, Jiangsu Province and Chinese Academy of Sciences, Nanjing, China; c The Jiangsu Provincial Platform for Conservation and Utilization of Agricultural Germplasm, Nanjing, China

**Keywords:** *Ulmus parvifolia*, chloroplast genome, phylogenetic analysis

## Abstract

*Ulmus parvifolia* is a promising tree species for landscaping. In this study, the complete genome of *U. parvifolia* was reported using next-generation sequencing technology. The chloroplast genome was a circular double-stranded DNA molecule with 159,182 bp in length. It contained a large single copy (LSC) region of 87,838 bp, a small single copy (SSC) region of 18,750 bp, and two inverted repeat (IRa and IRb) regions of 26,297 bp each, which exhibited a typical quadripartite structure. A total of 133 genes were identified, including 84 protein-coding genes, 41 tRNA genes, and eight rRNA genes. The overall GC content in the chloroplast genome was 35.59%. Phylogenetic analysis indicated that *U. parvifolia*, as a representative of Sect. *Microptelea* within the *Ulmus* genus, is sister to the species of Sect. *Ulmus*.

*Ulmus parvifolia*, the alternate name Chinese elm, is a promising landscape species in family Ulmaceae with a wide geographic distribution. It exhibits outstanding adaptability to a diverse range of adverse environmental conditions. It has been proven to be highly resistant to elm leaf beetle and Dutch elm disease (Bosu and Wagner 2007). In the meantime, *U. parvifolia* is recognized as having drought, heat, and cold resistance traits (Lyu et al. 2020). Nowadays, it is commonly planted on lawns, along streets, and in parks, which is ecologically important (Thakur and Karnosky 2007). Nevertheless, very limited genomic data is available on this species. Chloroplast genome is characterized by small genome size, maternal transmission, and low mutation rate, which make the chloroplast-derived markers suitable for species identification, phylogenetic analysis, and population genetics studies (Li et al. 2019; Cai et al. 2020; Mo et al. 2020). In this study, the complete chloroplast genome of *U. parvifolia* was *de novo* assembled, annotated, and analyzed phylogenetically.

Fresh leaves were collected from experimental farm of Jiangsu Academy of Forestry (Nanjing, China 118°45′57.30″E, 31°51′27.94″N) and were deposited in the Herbarium of Jiangsu Academy Forestry (JAF: Lyu20200512-3). Genomic DNA was extracted using Universal Plant Total DNA Extraction Kit (BioTeke, Beijing, China) according to the manufacturer's instruction. A paired-end library with approximate insert lengths of 300 bp was constructed by an Illumina Hiseq library kit (Illumina, San Diego, USA), and then the paired reads were sequenced on the Illumina Hiseq 4000 platform. *De novo* assembly and annotation of the chloroplast genome were conducted using NOVOPlasty and DOGMA, respectively.

The length of the entire chloroplast genome of was 159,182 bp. Its genome has a conserved quadripartite structure, consisting of 87,838 bp large single copy (LSC) region, 18,750 bp small single copy (SSC) region, and two 26,297 bp inverted repeat (IRa and IRb) regions. The chloroplast genome was predicted to make up of 133 genes: 84 protein-coding genes, 41 tRNA genes, and eight rRNA genes, of which, seven protein-coding genes, nine tRNA genes, and four rRNA genes were duplicated in the IR regions. The containing GC content in the chloroplast genome of *U. parvifolia* was 35.59%, and the corresponding values for the LSC, SSC, and IR regions were 33.05%, 28.56%, and 42.35%, respectively.

To determine the phylogenetic status of *U. parvifolia*, a phylogenetic tree was built using IQ-tree software (Nguyen et al. 2015) with the maximum-likelihood algorithm based on the chloroplast genome sequences of 16 representatives of Ulmaceae. Complete chloroplast genome sequence alignment was carried out using MAFFT program (Katoh and Standley 2013). The phylogenetic analysis showed that the seven species of *Ulmus* genus formed a paraphyletic clade with high support (bootstrap value 100%). Based on sectional-level taxonomic system, the genus *Ulmus* could be divided into four sections: Sect. *Ulmus*, Sect. *Microptelea*, Sect. *Blepharocarpa*, Sect. *Trichoptelea* and Sect. *Chaetoptelea* (Zhang et al. 2018). *Ulmus parvifolia* is a representatives of Sect. *Microptelea*, and our analysis revealed that it is sister to the species of Sect. *Ulmus* within the *Ulmus* genus. The chloroplast genomic resources reported here will provide a basis for future studying the phylogeny, genetic diversity, and adaptation of *U. parvifolia* (Figure 1).

**Figure 1.**
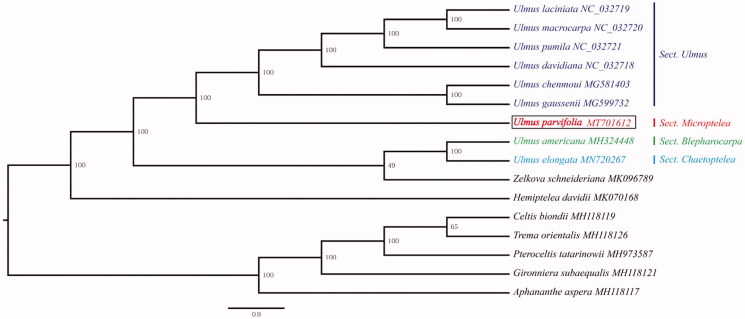
Phylogenetic tree construction using maximum-likelihood (ML) based on the 16 complete chloroplast genome sequences of Ulmaceae. The bootstrap support values were shown at the branches.

## Data Availability

The data that support the findings of this study are openly available in NCBI at http://www.ncbi.nlm.nih.gov/, reference number MT701612.
